# Molecular Identification and Polymorphism Determination of Cutaneous and Visceral Leishmaniasis Agents Isolated from Human and Animal Hosts in Iran

**DOI:** 10.1155/2013/789326

**Published:** 2013-10-28

**Authors:** Homa Hajjaran, Mehdi Mohebali, Setareh Mamishi, Farzaneh Vasigheh, Mohammad Ali Oshaghi, Saied Reza Naddaf, Aref Teimouri, Gholam Hossein Edrissian, Zabiholah Zarei

**Affiliations:** ^1^Department of Medical Parasitology and Mycology, School of Public Health, Tehran University of Medical Sciences, Tehran 14155 6446, Iran; ^2^Center for Research of Endemic Parasites of Iran (CREPI), Tehran University of Medical Sciences, Tehran, Iran; ^3^Pediatric Infections Research Center, Tehran University of Medical Sciences, Tehran, Iran; ^4^Department of Medical Entomology and Vector Control, School of Public Health, Tehran University of Medical Sciences, Tehran, Iran; ^5^Department of Parasitology, Pasteur Institute of Iran, Tehran, Iran; ^6^School of Public Health, Meshkin-Shahr Research Station, Tehran University of Medical Sciences, Iran

## Abstract

Amplification of internal transcript spacer 1 of ribosomal RNA (ITS1-RNA) gene followed by RFLP analysis and sequencing was used to identify the causing agents of cutaneous and visceral leishmaniasis (CL and VL) in humans and animal reservoir hosts from various geographical areas in Iran. We also used random amplified polymorphic DNA (RAPD-PCR) to obtain polymorphisms among isolates of *Leishmania* spp. Totally, 362 suspected human and animal cases including 173 CL, 49 VL, 60 rodents, and 80 domestic dogs were examined for *Leishmania* infection. From 112 culture-positive samples prepared from CL cases, 75 (67%) were infected with *L. major* and 37 (33%) with *L. tropica*. Of the 60 rodents examined, 25 (41.6%) harbored the *Leishmania* infection; 21 were infected with *L. major* and 4 with *L. turanica*. From 49 suspected VL, 29 were positive by direct agglutination test (DAT), whereas microscopy detected parasite in bone marrow of 25 and culture in 28 of the patients. Two VL patients were infected with *L. tropica* and 26 with *L. infantum*. Of the 80 domestic dogs, 56 showed anti-*Leishmania* antibodies with DAT. Of these, 55 were positive by both microscopy and culture. Molecular identity, obtained only for 47 samples, revealed *L. infantum* in 43 and *L. tropica* in 4 dogs. The polymorphisms among *L. tropica* and *L. major* isolates were 3.6% and 7.3%; the rate among human and canine VL isolates was 2.8% and 9.8%, respectively. Our results showed that at least four different *Leishmania* species with various polymorphisms circulate among humans and animal hosts in Iran.

## 1. Introduction

Leishmaniases are a group of diseases caused by the parasites belonging to the genus *Leishmania*. The disease affects 98 tropical and Mediterranean countries, and 1.5 to 2 million cases are estimated to occur annually worldwide, with up to 350 million people at risk of infection [1]. Leishmaniases present a wide spectrum of clinical manifestations including cutaneous (CL), diffuse cutaneous (DCL), mucocutaneous (MCL), and potentially fatal visceral leishmaniasis (VL), also known as “Kala-Azar,” that are largely caused by different *Leishmania *species. In old world, three species caused CL: *L. tropica, L. major* (both usually cause localized skin lesions), and *L. aethiopica* that cause nonulcerative disseminated cutaneous lesions. VL is caused by parasites of the *L. donovani-L. infantum* complex. Recently, *L. tropica* has been implicated in several human cases with VL manifestations [2]. In Iran, the etiological agents of anthroponotic CL (ACL) and zoonotic CL (ZCL) are known to be *L. tropica *and* L. major, *respectively. *L. infantum,* the principal agent of VL, causes splenomegaly and hepatomegaly [3]. Most of ACL are reported from northeastern and central parts of Iran [4–6], while ZCL extends from northeast to center and west of Iran and covers all southern provinces as well [7–9]. More than 90% of the visceral cases in Iran are reported from the northwest and some southern provinces including Fars and Bushehr [10]. All the three species of *Leishmania *could be found in some areas like Fars province in the south [11]. In Iran, the main reservoir hosts for ACL are human infected patients, for ZCL are some of the desert rodents particularly the great gerbils (*Rhombomys opimus*), and for VL are domestic and wild canines (mainly domestic dogs) [4, 12–15]. In addition to the classic clinical leishmaniasis manifestations, there is a variety of atypical signs in CL including lupoid, mucosal lesions, and disseminated forms and in VL including symptomatic and asymptomatic forms [16–18]. The diagnosis of clinical forms of leishmaniasis is commonly based on visualization of the parasite in Giemsa-stained smears by microscopy and culture. Despite sensitivity and specificity, such methods fail to identify the infecting *Leishmania* species [19, 20]. Over the last two decades, DNA-based approaches including PCR followed by sequencing or RFLP analysis and species-specific PCR have been widely used for the identification of *Leishmania* species [20–23].

Our study aimed to identify different *Leishmania *species in some foci of Iran and obtain polymorphisms among the species using RAPD-PCR.

## 2. Materials and Methods

### 2.1. Sampling

#### 2.1.1. Cutaneous Leishmaniasis

Lesion aspirates were obtained from 173 suspected CL patients referred either to leishmaniasis laboratory at the School of Public Health, Tehran University of Medical Sciences (TUMS), or district health centers (DHCs) and hospitals in various regions of Iran from 2004 to 2012. Most patients were from northern, northeastern, western, central, and southern areas of the country. Samples were collected by injecting 0.2 mL of sterile saline into the dermis of the internal border of skin lesions with sterile insulin syringes. After suction, the sample was transferred to RPMI-1640 medium culture and some portion was checked for *Leishmania* infection by microscopy following the preparation of Giemsa-stained smears [22]. 

#### 2.1.2. Rodents

Sixty rodents including 36 *Rhombomys opimus*, 5 *Meriones libycus*, 13 *Nesokia indica*, 5 *Tatera indica*, and one *Mus musculus* were trapped by live baits traps from CL endemic areas in the northeast and west of Iran. None of the animals had any acute cutaneous lesion. For preparation of samples, the external edges of the ear lobes were cut with scissors after washing and disinfection, and then low amounts of the exudates were transferred to culture media [24]. Giemsa-stained smears were prepared from the same samples. 

#### 2.1.3. Visceral Leishmaniasis

Blood samples were taken from 49 suspected VL cases, presented with fever, weakness, and hepatosplenomegaly, at the medical health centers or leishmaniasis laboratory at the School of Public Health (SPH), TUMS. Most patients were from northwestern, western, central, and southern areas of the country. Also, the bone marrow aspirate was taken from iliac of the same patients under local anesthesia by physicians. 

Also, blood samples were taken from eighty dogs living in VL endemic area in the northwest and localities in the northeast and center of the country. Of the 80 dogs, 59 presented one or several of the typical clinical signs (such as lymphadenopathy, hair loss, dermal lesions, onychogryposis, and cachexia) and 56 had showed anti-*L. infantum* antibodies. All of the 59 dogs with typical clinical signs were sacrificed after obtaining the consent of their owners, and Giemsa stained- smears were prepared from liver and spleen tissues.

Peripheral blood samples were collected into tubes with sodium citrate anticoagulant 4% (Merck, Germany) for PCR testing and into tubes without anticoagulant for DAT. All samples used for serology and PCR were stored at −20°C until use. 

All experiments on the humans and animals were performed according to the guidelines of the Ethical Board of Tehran University of Medical Sciences, Iran.

#### 2.1.4. Parasite Culture and Cryopreservation

All the specimens including exudates from human skin lesions and rodents ears, aspirates from bone marrow of human patients, and liver and spleen of dogs were cultured in RPMI-1640 medium (Gibco, Life technologies GmbH, Frankfurt, Germany) supplemented with 10–15% fetal bovine serum (Gibco, Germany), 100 U/mL penicillin, and 100 ug/mL streptomycin (Gibco, Germany) and incubated at 24-25°C. Five days later the last subcultured parasites were harvested, washed in sterile phosphate buffered saline (PBS, pH: 7.2–7.4), and kept in −20° until used. All the cultures specimens were preserved in liquid nitrogen for further possible use. 

#### 2.1.5. Preparation of DAT Antigen and Performance of the Test

*L. infantum* (MCAN/IR/07/Moheb-gh; GenBank accession number FJ555210) was cultured in RPMI1640. The parasites were harvested at early stage of stationary phase (about 120 h later), washed with PBS (pH = 7.2), and subjected to trypsin digestion. The parasites were stained with Coomassie brilliant blue R-250 dye (Sigma, USA), fixed with formaldehyde (Merck, Germany) 1.2%, and used for DAT on humans, and dogs' sera. DAT was performed to determine the presence of the anti-*Leishmania* antibodies [14, 25, 26]. The titers ≥ 1 : 3200 and ≥320 were considered positive for humans and dogs, respectively [10]. 

#### 2.1.6. DNA Extraction, PCR, and RFLP

DNA was extracted from cultured parasites from human skin lesions, rodents' ears exudates, and whole blood samples of suspected VL patients and canine visceral leishmanissis dogs by using the High Pure PCR Template Preparation Kit (Roche Diagnostics GmbH, Mannheim, Germany) according to the manufacturer's instructions. Partial ITS1 of ribosomal RNA (ITSI-rRNA) gene of the parasites was amplified using the primers LITSR (5′-CTGGATCATTTTCCGATG-3′) and L5.8S (5′-TGATACCACTTATCGCACTT-3′) and PCR conditions outlined by others [19]. Amounts of 10 *μ*L of amplicons were digested with Fast digestion HAEIII (BsuRI) enzyme (Fermentas GmbH, Thermo Scientific, Germany) according to the manufacturer's instructions, and the fragments were visualized on 3% agarose gels [22]. *Leishmania* species were identified based on obtained patterns alongside reference species including *L. tropica* (GenBank accession number EF653267), *L. major* (GenBank accession number JN860745), *L. turanica* (GenBank accession number EU395712), and *L. infantum* (GenBank accession number EU810776).

#### 2.1.7. Variation Analyses by RAPD-PCR

RAPD-PCR was performed for 46 DNA extracted from cutaneous and visceral cultured parasites samples with the primer AB1-O7:5′-GGTGACGCAG-3′ and PCR conditions outlined by others [4, 27]. This primer was chosen due to its reproducibility and its ability to reveal inter- and intraspecies variation. 

**Figure 1:**
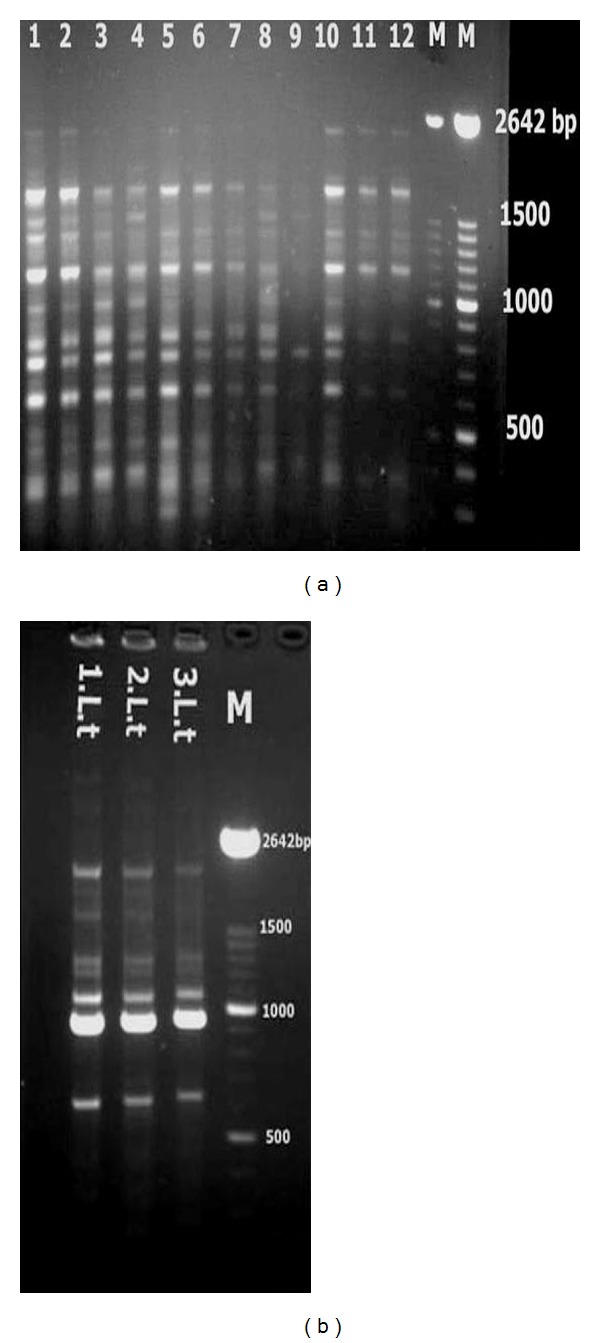
(a) Random amplified polymorphic DNA (RAPD) profiles obtained from *Leishmania *stocks and isolates with the AB1-O7 primer. Lane 1, *L. infantum* stock, lanes 2–12, *L. infantum isolates*. (b) Lane 1, *L. tropica* stock, lanes 2-3, *L. tropica*. M: 100 bp size marker (Roche).

Variations among isolates of different species were calculated based on profile of bands produced by AB1-O7 primer (Figure 1). A matrix for each of the isolates was constructed with the numbers “1” and “0” corresponding to the presence and absence of each possible band. For determining the polymorphism rate among different *Leishmania* species, we identified the bands that were common in less than 50% of the isolates (polymorphic bands) using the following equations, where *Z* was calculated by (1) for each polymorphic band. *Z*-test was used for estimating the relative risk between two independent groups [28, 29]. If the obtained value for *z* in a band is less than <1.64 with a confidence interval of more than 95% (*P* < 0.05), then it will be included as a polymorphic band in the matrix. After determining the polymorphic bands, polymorphism rate was calculated via (2), where *X* is the number of isolates which contain a polymorphic band, *N* is the total number of isolates in each group, and *M* is the number of total bands in each isolate

(1) $$\begin{array}{c} Z=\frac{\left(X/N\right)-0.5}{\sqrt{\left(0.5\times 0.5\right)/N}}, \end{array}$$

(2) $$\begin{array}{c} \text{Polymorphism}\;\text{rate}=\frac{X}{M\times N}\times 100. \end{array}$$

#### 2.1.8. Nucleotide Sequence and Phylogenic Analyses

Partial sequences of ITS1 gene from 20 cutaneous and visceral *Leishmania* isolates (Table 1) were sequenced using LITSR as the forward primer. The sequences were aligned by ClustalX software, the similarities among them were calculated, and a phylogenic tree was constructed by using Tamura 3-parameter option of the maximum likelihood method by MEGA 5 software.

## 3. Results

### 3.1. Cutaneous Leishmaniasis

#### 3.1.1. Human

**Figure 2:**
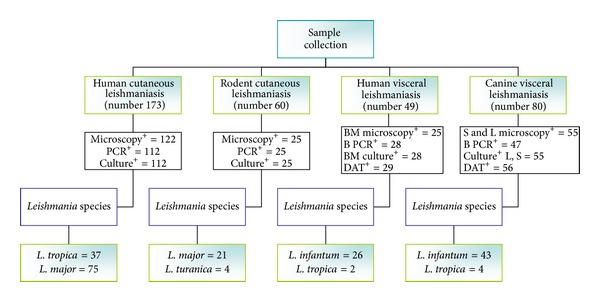
Flow chart which outlines the major parts of the study and results. B PCR: blood PCR, BM: bone marrow, S: spleen tissue, L: liver tissue, and DAT: direct agglutination test.

**Figure 3:**
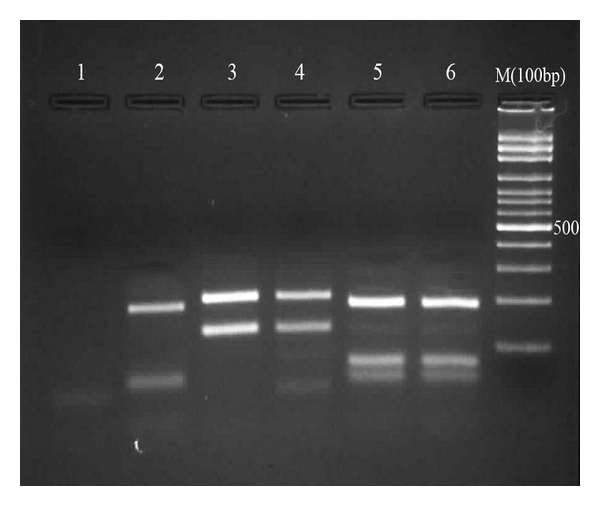
Restriction fragment length polymorphism (RFLP) patterns obtained from *Leishmania *stocks and patients samples. lane 1, negative control, lane 2, *L. tropica*, lanes 3, 4, *L. major*, lanes 5,6, *L. infantum. *M: 100-bp size marker (Fermentas).

**Figure 4:**
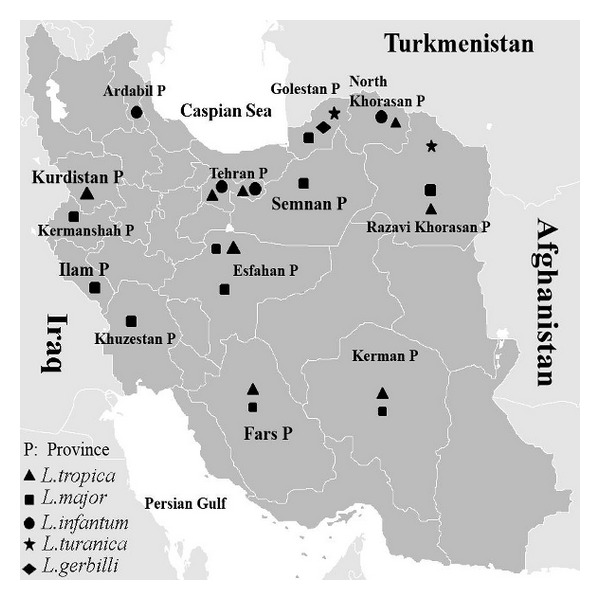
Distribution of *Leishmania* species isolated from human and animal hosts from some parts of Iran, identified based on ITS1-PCR-RFLP (2004–2012).

 Among 173 suspected CL patients, 122 (70.5%) were positive by microscopy and 112 (64.7%) by culture method (Figure 2). DNA extracted from 112 culture positive samples yielded an amplicon of 300–350 bp. Digestion of amplicons with HAEIII (BsuRI) produced two profiles: 75 amplicons (67%) produced two bands of 220 bp and 140 bp indicative of *L. major*, and other 37 (33%) produced three bands of 200 bp, 60 bp, and 50 bp indicative of* L. tropica *(Figure 3). Almost all of the *L. tropica* isolates originated from northeastern areas, while *L. major* isolates belonged to the center, west, southern, and southwest of the country (Figure 4).

#### 3.1.2. Rodents

Of the 60 caught rodents, 25 (24 *R. opimus* and one *N. indica*) were parasitologically positive (microscopy and/or culture). Among the infected *R. opimus,* 21 harbored *L. major* and 3 *L. turanica*. The infecting species in *N. indica *was *L. turanica *(Figure 2).

### 3.2. Visceral Leishmaniasis 

#### 3.2.1. Human

From 49 suspected VL patients, 29 (59.2%) had anti-*Leishmania* antibodies and were considered positive (titers ≥ 1 : 3200). Microscopy and culture methods identified *Leishmania* infection in 25 (51.02%) and 28 (57.14%) individuals, respectively. ITS1-PCR using the DNA from 49 patients' blood yielded the expected band only in 28 cases (57.14%). Digestion of amplicons with HAEIII (BsuRI) yielded three bands of 200 bp, 80 bp, and 60 bp in 26 (92.86%) isolates indicative of *L. infantum* and three bands of 200 bp, 60 bp, and 50 bp in 2 (7.14%) isolates indicative of *L. tropica *(Figure 3).

#### 3.2.2. Domestic Dogs

 Analyses of sera from 80 domestic dogs showed that 56 (70%) were positive (at titers ≥ 1 : 320). Whole blood dog DNA samples were successfully amplified in only 47 (58.75%) cases. RFLP analyses revealed *L. infantum* in 43 dogs (91.5%) and *L. tropica* in 4 dogs (8.5%). Of the 59 dogs with typical symptomatic VL signs (including the 56 DAT serologically positive cases), 55 showed infection in spleen and liver with both microscopy and culture (Figure 2).

### 3.3. RAPD-PCR and Polymorphism Rate

 The polymorphism rate among *L. tropica *isolates was 3.6%. The rate among *L. major *isolates was 7.3% and among human VL isolates was 2.8%. The highest rate (9.8%) was found among canine VL isolates.

### 3.4. Phylogenetic Tree

**Figure 5:**
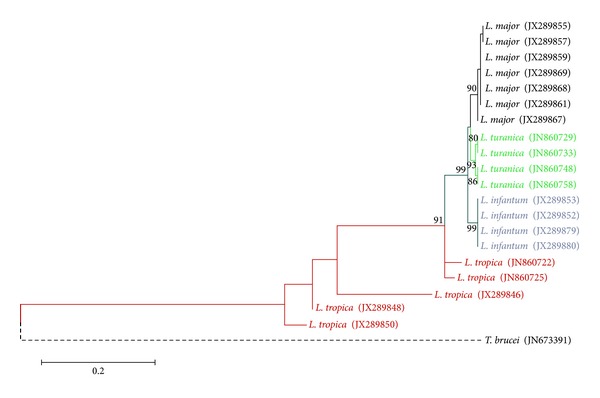
Phylogenic tree of the ITS1 region nucleotide sequences of *Leishmania *isolates recovered from humans and reservoir animals infected with cutaneous and visceral leishmaniasis. The tree was constructed by using the maximum likelihood, Tamura 3-parameter model in MEGA software version 5. The numbers above the branches indicate the percentage of bootstrap. Branches without numbers have frequencies of less than 70%.

The *Leishmania* isolates were grouped into four main clads representing *L. major*, *L. turanica*, *L. infantum,* and *L. tropica*. Within the clad, *L. major* was more associated with *L. turanica* than *L. infantum*. The *L. tropica* supports a clear divergence between *L. tropica* isolates from the other three species. The numbers above the branches indicate the percentage of bootstrap samplings. There was no clear grouping among the 20 isolates according to their geographical origin (Figure 5).

Details of the specimens sequenced and submitted to GenBank are shown in Table 1.

## 4. Discussion

In this study, we could identify four species of *Leishmania* including *L. major, L. tropica*, *L. infantum*, and *L. turanica* by ITS1 gene followed by RFLP and sequencing. The molecular results on the *Leishmania* isolated from human cutaneous lesions are consistent with the epidemiologic studies committed in recent decades [3]. The results showed the major causes of CL in Iran were *L. major *(67%) and *L. tropica* (33%). The major foci of ZCL transmission were in the northern (Golestan), northeastern near Turkmenistan's border, central (Esfahan), western (Kermanshah) near the Iraqi border, and southern (Khuzestan) provinces of Iran. The major foci of ACL transmission were in the northeast (Razavi Khorasan province) and center of Iran in the city of Bam (Kerman province) especially after the earthquake in 2003.

*L. major *was found to be the most prevalent species in *R. opimus* (the main reservoirs of ZCL) followed by *L. turanica* as the second agent [13]. *L. turanica* is incapable of infecting humans, but it causes lesions in laboratory animals [30]. 

In suspected VL patients, DAT showed presence of anti-*L. infantum *antibodies in 29 (59.2%) of the human cases. PCR detected *Leishmania *DNA in blood samples of 28 (57.14%) individuals. RFLP analyses revealed *L. infantum* as the main causative agent of VL (92.86%) and* L. tropica* as the secondary agent (7.14%). This finding is in agreement with the results of other studies performed in Iran [10, 16]. The main endemic areas of VL were Ardebil province (Meshkin-Shahr district) in the northwest and Fars provinces in the south. HIV-*Leishmania *coinfection has been reported recently from northeastern Iran [31].

 Of the 49 bone marrow aspirates from human VL, 25 (51.02%) showed amastigote forms of *Leishmania* sp. by microscopy. DAT and PCR were both positive in all the 25 human patients.

In a similar study, PCR and microscopy of bone marrow aspirates were shown to be equally sensitive in patients with microscopically confirmed VL. Moreover, PCR could detect *Leishmania* DNA in bone marrow aspirates in 66.7% of suspected VL patients, while microscopy of the same material was negative [32].

Serology was more sensitive than PCR in diagnosis of suspected VL dogs. As in humans, *L. infantum* was the main causing agent of VL in dogs (91.5%) and *L. tropica* was found in 8.5% of the cases. One of the important findings of this study was molecular detection of *L. tropica* DNA in a considerable number of humans and dogs with VL.

In a similar study, microscopy detected parasite in liver and spleen of 93.2% of symptomatic dogs, while serology was positive in 94.9% and PCR in 76.6% of cases [17]. PCR seems to be less sensitive than microscopy or serology for the detection of *Leishmania* in blood of suspected VL dogs [33]. Phylogenic trees derived from the ITS1 sequences support a clear divergence between *L. tropica* from the other three species, but there was no clear grouping among the isolates according to their geographical origin. In RAPD-PCR assay, we used the primer AB1-07, which had the most consistency rate between different populations of *Leishmania* strains in different regions. 

## 5. Conclusions

Characterization of *Leishmania *isolates collected from different parts of Iran showed that *L. tropica *with 3.6% polymorphisms was the primary agent in the ACL foci and *L. major* with 7.3% genetic variations was the predominant agent in the ZCL areas of Iran. Moreover, *L. infantum* with 2.8% genetic variations in human VL and 9.8% polymorphisms in canine VL was found in VL endemic areas of Iran. The results of this study showed that PCR-RFLP and RAPD-PCR, despite some limitations, are simple and powerful tools for the characterization and determination of *Leishmania *species polymorphisms.

## Figures and Tables

**Table 1:** The accession numbers of *Leishmania *ITS1 sequences in Iranian species submitted in GenBank.

No.	Code	Place of isolation city/province	Disease	Source	RFLP/SEQ	Species	ACC.NO
1	MHOM/IR/10/Esfahan-1	Esfahan/Esfahan	CL	Human	+/+	*L. major *	JX289855
2	MHOM/IR/10/Esfahan-3	Esfahan/Esfahan	CL	Human	+/+	*L. major *	JX289857
3	MHOM/IR/10/Esfahan-5	Esfahan/Esfahan	CL	Human	+/+	*L. major *	JX289859
4	MHOM/IR/10/Esfahan-7	Esfahan/Esfahan	CL	Human	+/+	*L. major *	JX289861
5	MHOM/IR/05/Kermanshah_kr3	Kermanshah/Kermanshah	CL	Human	+/+	*L. major *	JX289869
6	MHOM/IR/05/Kermanshah_kr1	Kermanshah/Kermanshah	CL	Human	+/+	*L. major *	JX289867
7	MHOM/IR/05/Kermanshah_kr2	Kermanshah/Kermanshah	CL	Human	+/+	*L. major *	JX289868
8	MHOM/IR/12/Bam2	Bam/Kerman	CL	Human	+/+	*L. tropica *	JX289846
9	MHOM/IR/12/Bam4	Bam/Kerman	CL	Human	+/+	*L. tropica *	JX289848
10	MHOM/IR/12/Bam6	Bam/Kerman	CL	Human	+/+	*L. tropica *	JX289850
11	MHOM/IR/02/M12-Khorasan	Mashhad/Khorasan	CL	Human	+/+	*L. tropica *	JN860722
12	MHOM/IR/02/M39-Khorasan	Mashhad/Khorasan	CL	Human	+/+	*L. tropica *	JN860725
13	MRHO/IR/11/GOL-5	Golestan	CL	Rodent	+/+	*L. turanica *	JN860748
14	MRHO/IR/10/Khorasan6	Khorasan	CL	Rodent	+/+	*L. turanica *	JN860733
15	MRHO/IR/11/GOL-19	Golestan	CL	Rodent	+/+	*L. turanica *	JN860729
16	MRHO/IR/11/GOL-15	Golestan	CL	Rodent	+/+	*L. turanica *	JN860758
17	MCAN/IR/08/Moshfe237-VL-8	Meshkinshahr/Ardebil	VL	Canine	+/+	*L. infantum *	JX289879
18	MCAN/IR/08/Moshfe240-VL-9	Meshkinshahr/Ardebil	VL	Canine	+/+	*L. infantum *	JX289880
19	MCAN/IR/11/Zare	Bomehen/Tehran	VL	Canine	+/+	*L. infantum *	JX289852
20	MCAN/IR/11/Ziaee	Meshkinshahr/Ardebil	VL	Canine	+/+	*L. infantum *	JX289853
